# Editorial: Physical time within human time

**DOI:** 10.3389/fpsyg.2023.1293198

**Published:** 2023-10-24

**Authors:** Anne Giersch, Leonardo P. G. De Assis

**Affiliations:** ^1^Université de Strasbourg, Strasbourg, Alsace, France; ^2^INSERM U1114, Institut National de la Santé et de la Recherche Médicale (INSERM), Strasbourg, France; ^3^Department of Psychiatry, University Hospital of Strasbourg, Strasbourg, Alsace, France; ^4^Stanford Pre-Collegiate University-Level, Stanford University, Redwood City, CA, United States; ^5^School of Humanities and Liberal Studies, San Francisco State University, San Francisco, CA, United States

**Keywords:** psychological time, physical time, present time, block universe, time flow, quantum physics

Diving in the Research Topic “*Physical time within human time”* means giving up our intuitions about what time is. The notion of a singular, continuous flow of time has come under scrutiny in both physics and neuroscience, creating a challenge in reconciling perspectives across these research domains. Physics debates the very existence of time itself, questioning the presence of a past, present, and future in contrast to an eternal block universe.

Indeed, some physicists argue that the block universe model, which implies a timeless cosmos, can still explain the perception of the passage of time. They argue that the physicist's task is to describe how the universe appears from the point of view of individual observers. On the other hand, others argue that the passage of time is physical and that the future does not exist ontologically. They believe that the task of physics is to explain not just how time seems to pass, but why. There are also physicists who propose alternative models of time, such as the idea that gravity, not thermodynamics, points the arrow of time, or that time is a fundamental feature of the cosmos that emerges naturally from the structure of space time. But amid this variety of views with different models of time in physics, we see that there is an interest among physicists to understand why the models they build conflict with the human perception of time.

Neuroscience, including psychology, offers a distinct perspective on the concept of time, contrasting with the view in physics. Unlike physics, neuroscience explores the experience and perception of time. Neuroscientists recognize that our understanding of time is intertwined with cognitive processes and individual perspectives. They study how people perceive, process, and remember time, and the temporal dynamics of brain activity, cognition and behavior. By focusing on the psychological and neurobiological dimensions, neuroscientists reveal the complexities of human temporal experiences, which can vary across individuals, contexts, and cultures. Those questions open the door to a multitude of theoretical possibilities, that entail, or not, a reconciliation of the physical and psychological views of time.

We believe it's essential to consider a wide range of perspectives to understand the potential consequences of the new concepts of timing and to test these ideas. This Research Topic precisely offers that. The two papers by Buonomano and Rovelli (2022), as well as the modified IGUS model by Gruber et al. (2022), illustrate the issues we are facing, and the commentaries underline the difficulties encountered when trying to reconcile the physical and the neuroscientific view of time. Moreover, they reflect the diversity of the views and problems elicited by the proposed solutions. Considering that various authors have explored related questions with diverse approaches and thematic focuses, we have structured this editorial into distinct topics.

## What is veridical and what is illusory? Our subjective experience is reflecting something veridical?

Many commentaries emphasize the difficulty to distinguish between what is veridical and illusory. However, whereas some authors emphasize the importance of taking physics into consideration, others emphasize the importance of our subjective experience to define time ontology. Dorato remarks that it can be difficult to distinguish between what would be attributed to physics vs. psychology: what is “information” in physics, and how should we qualify the “illusory” output of a robot? He adds that time travel is not accounted for by e.g. IGUS, but also acknowledges that naive physics may not be the best choice to access time ontology.

Turning our focus to the subjective experience, the following contributors delve even deeper into this aspect.

Arstila acknowledges the merits of the proposed models but still asks whether it is really the case that any temporal component has two aspects, veridical and illusory, as proposed in the dualistic model of Gruber et al. (2022). Like several authors he questions whether the snapshot theory should be seen as veridical and the specious present as an illusion.

Dainton expresses dissatisfaction with labeling the snapshot theory as veridical, emphasizing the challenge in defining the present moment (3 seconds or less). He reminds us that any theory should account for our phenomenological experience of continuity and sense of present.

Wittmann also counters the claim made by Gruber et al. (2022) that the present moment and dynamic change are illusory. Wittmann asserts the reality of the present moment and argues that our perception of time reflects the temporal structure of the world. The article delves into the neurological and philosophical implications of time perception, emphasizing the importance of perceiving the dynamic passage of events for proper functioning. Wittmann explores the concept of phenomenal consciousness as a distinct experience within the continuous flow of time. Overall, the article challenges the idea that the present moment is an illusion and highlights its significance in our perception and experience.

Elliott investigates the concept of time from philosophical and scientific perspectives, tracing its historical origins and metaphysical implications according to Aristotle's interpretation. He argues that although experienced time is real, its dimensionless nature prevents its operational use in physics. He further insist that time processing in the brain does not necessarily lead to a conscious experience. Elliott suggests a broader understanding of temporal experience, acknowledging the challenge the distinction between physical and psychological time poses to reductionist science.

Miller and Wang question the subjective experience itself. They dive deeply in the topic of presentness by rejecting the idea that our experience of flow is one of a changing present. They similarly question the concept of self persistence, and this leads them to doubt that the experience of flow is an illusion.

Shifting our perspective away from exploring subjective time as we experience it, certain authors raise thought-provoking suggestion whether we should question those experiences.

## What is veridical and what is illusory: should physics lead us to change the way we understand time?

Prosser delves into the intricate connection between time, experience, and neuroscience, cautioning against adopting a neuroscientific perspective that contradicts established principles in physics. He emphasizes the need for caution when drawing conclusions about the nature of time solely from subjective experience.

Glicksohn not only questions the fact that the passage of time is illusory, but also the linearity of the passage of time. After discussing how the passage of time can be explored, he suggests we should question the discontinuity and not only the continuity of time. As a matter of fact, since the time problem comes from an apparent contradiction between a frozen time in physics and the passage of time in psychology, an alternative is of course to question those statements.

Farr argues that the main problem lies between the time of experience and commonsense time, rather than between physics and the time of experience. He prompts us to reconsider whether quantum physics describes a static world and challenges the notion of our experience of time as one of flow.

Silberstein presents his argument that time is a relational property of beings with bodies, rather than a property projected by the brain. He supports a Jamesian form of neutral monism, which posits that the mental and the physical are neutral and not separate entities. According to this perspective, physics is rooted in and influenced by subjective experience. Silberstein also criticizes the primary/secondary distinction, asserting that the world cannot be neatly divided into categories such as physical/mental or subject/object. Additionally, he challenges the notion of consciousness as qualia, suggesting that intrinsic physical properties should be replaced by qualitative aspects such as qualia or subjectivity.

If defining timing is already difficult, the two papers by Buonomano and Rovelli (2022) and Gruber et al. (2022) also address the question of the link that can be made between the concepts of time in physics and psychology. Some contributors explore how this question can be investigated.

## What is veridical and what is illusory: how should we use VR? Motion and change, and psychophysics as a link between psychology and physics?

Latham and Holcombe question how we can explore the experience of time in psychology. More specifically they question the way (Gruber et al., 2022) test the possibility for participants to experience a past event as being a re-experienced present. They further stress the possibility that those participants have a vivid experience while knowing they are re-experiencing a past event. They propose some additional and interesting means to test this idea, using affordances.

Huggett, Deng, and Balcells all take the example of motion and change, and like the majority of authors, question what is illusory and what is veridical.

Balcells suggests that some changes can be described by physics and may indeed be veridical, while Deng suggests that “becoming” might be the illusory part of veridical change. Huggett proposes that motion and the sense of flow are not illusions but rather misinterpretations of perceptual information, which challenges the proposed models.

Grondin brings to our attention the objective of psychophysics, which is to define the relationship between the outer world and our perception of this world. He underlines temporal laws as old as the Weber fraction do not hold beyond some durations, as if reflecting a disruption of the flow of time: our perception of the world is constrained by the way the brain works, and our senses serve as evidence for the world's existence.

Embarking on the journey of reconciling perspectives on time in physics and psychology, the following authors explore the theoretical questions that arise from this endeavor.

## Reconciling the manifest image of time with its scientific image?

Several authors discuss to which extent physical laws can be reconciled, or embedded in the psychological experience.

Balashov questions whether it is possible to “reconcile the manifest image of time” with its scientific image. He reminds us of the stage theory, in which objects are themselves states and are thus temporary. This leads however to a special role for the present, which may or may not be inconsistent with a physical view. The sense of present time plays an important role in many comments.

In his article, Dieks suggests that the core elements of human time can be found at a fundamental physical level. He proposes that quantum mechanics may provide a physical counterpart to the subjective nature of human time. He argues that temporal relations similar to those governing our experiential time might exist in fundamental physical systems, establishing a connection between quantum mechanics and human time.

Romero explores the distinction between physical time and psychological (perceptual) time, highlighting that they arise from the same underlying physical laws. Rather than being a passive response to sensory input, the experience of time is actively generated by the brain's predictive processes and the body's sensorimotor activities. Differences in how we perceive time are relative and reflect variations in the distribution of properties within the four-dimensional spacetime framework. To test the notion of a time constructed by the brain, the author proposes manipulating the information presented to the brain through an information gathering and utilizing system (IGUS).

Paganini examines the nature of time by exploring the theoretical proposals of Buonomano and Rovelli (2022) and Gruber et al. (2022). Rather than directly comparing their cosmological theories, Paganini focuses on the philosophical challenges presented by each notion of illusion. Gruber et al. argue for the illusory nature of our temporal experience due to the Block Universe concept, while Buonomano and Rovelli (2022) propose that our perception of time may hold validity in relation to local reality. Paganini refrains from taking a position on the accuracy or validity of these notions, but encouraging critical engagement and contributing to the discourse on the nature of time.

In conclusion, the diverse contributions presented in this Research Topic of Theoretical and Philosophical Psychology journal have shed light on the complex nature of time and our perception of it. The authors have explored the boundaries between what is veridical and illusory, delving into the domains of physics, psychology, neuroscience, and philosophy. While there is ongoing debate regarding the nature of the present moment, the experience of flow, and the relationship between subjective and objective time, these discussions have enriched our understanding and prompted critical engagement with the concept of time. It is clear that a comprehensive exploration of the concept of time requires interdisciplinary collaboration, bridging the realms of philosophy and science. By continuing to investigate and challenge existing theories, we can hope to deepen our comprehension of time and its profound implications for our perception and experience.

## Author contributions

AG: Writing—original draft, Writing—review and editing. LD: Writing—review and editing.
